# ShenQiWan ameliorates renal injury in type 2 diabetic mice by modulating mitochondrial fusion and endoplasmic reticulum stress

**DOI:** 10.3389/fphar.2023.1265551

**Published:** 2023-11-10

**Authors:** Wei Xiong, Jing Feng, Yumeng Liu, Jiapeng Liu, Liang Fu, Qian Wang, Xia Li, Shuyu Li

**Affiliations:** School of Traditional Chinese Medicine, Beijing University of Chinese Medicine, Beijing, China

**Keywords:** diabetic nephropathy, ShenQiWan, type 2 diabetic mice, mitochondrial fusion disorder, endoplasmic reticulum stress

## Abstract

**Background:** ShenQiWan is commonly used in traditional Chinese medicine for the treatment of diabetic nephropathy, which is closely related to mitochondrial fusion and endoplasmic reticulum stress. This study aimed to investigate the intervention effect and molecular mechanisms of ShenQiWan on renal injury in KKAy mice.

**Methods:** C57BL/6J mice (11 weeks old) were fed a regular diet upon arrival, while KKAy mice (11 weeks old) were fed a high-fat diet upon arrival. At 12 weeks of age, KKAy mice with random blood glucose ≥13.9 mmol/L were identified as diabetic mice and randomly divided into the model group (*n* = 30) and the treatment group (*n* = 30), while C57BL/6J mice of 12 weeks old (*n* = 30) served as the control group. The treatment group received daily aqueous decoction of ShenQiWan (13.5 g/kg), while the control group and model group received daily equal amounts of saline from 12 weeks old to 24 weeks old. The general status of mice was observed regularly, and fasting blood glucose and 24-hour urine microalbumin were measured. Ten mice were euthanized in each group at the age of 16, 20, and 24 weeks, serum samples were used for biochemical indexes and kidney tissues were used for morphological studies. GRP78, OPA1, MFN1, MFN2 mRNA and protein expression were detected by Real-time PCR, immunohistochemistry and Western blot.

**Results:** The mice in the model group exhibited symptoms of lethargy, slow movement, obesity, polyuria and proteinuria. Morphological observation revealed pathological changes, including thickening of the glomerular basement membrane and interstitial fibrosis. After treatment with ShenQiWan, the fasting blood glucose level of KKAy mice was significantly reduced, urinary albuminuria was decreased, serum biochemical indexes were improved, renal tissue pathological changes were significantly alleviated. The results also showed a significant reduction in the expression of endoplasmic reticulum stress-related factor GRP78 and an increase in the expression of mitochondrial fusion-related factors OPA1, MFN1 and MFN2 after treatment with ShenQiWan.

**Conclusion:** ShenQiWan can protect diabetic mice from renal damage by modulating mitochondrial fusion and alleviating endoplasmic reticulum stress, exerting its protective effects.

## Introduction

Diabetic Nephropathy (DN) is the most severe diabetic microvascular complication, which is also a significant cause of end-stage renal disease (Umanath and Lewis, 2018; Bonner et al., 2020). Due to the complex development process of DN, its pathogenesis remains incompletely understood, making it a hot topic of research both domestically and internationally. The endoplasmic reticulum (ER) is an important organelle involved in protein synthesis, folding, modification and cell homeostasis. Various pathological factors can disrupt ER homeostasis and trigger endoplasmic reticulum stress (ERS) (Ni et al., 2021; Zhao et al., 2022). Numerous studies have demonstrated that ERS plays an essential role in the occurrence and progression of DN (Fan et al., 2017; Shahzad et al., 2020; Pontes-da-Silva et al., 2022). The kidney contains abundant mitochondria, second only to the heart in terms of quantity. Extensive research has also confirmed the importance of intact mitochondrial structure and function for renal health (Hallan and Sharma, 2016). Impaired mitochondrial function, leading to inadequate ATP supply and excessive generation of reactive oxygen species (ROS), is closely associated with the development of DN (Saxena et al., 2019).

DN falls under the category of “Xiao Ke disease” in traditional Chinese medicine. As one of the representative prescriptions of “Synopsis of the Golden Chamber,” ShenQiWan has the effect of nourishing essence, enriching the marrow, nourishing kidney yin and tonifying kidney yang, and has achieved therapeutic efficacy in the prevention and treatment of diabetic nephropathy (Hu et al., 2021). Previous studies by our research group have also demonstrated that ShenQiWan can alleviate the progression of diabetic nephropathy by influencing endoplasmic reticulum stress (Yumeng et al., 2022; Jiapeng et al., 2023). Currently, research on the treatment of DN with ShenQiWan primarily focuses on clinical efficacy observation, with limited basic research. Furthermore, studies on the mechanism of DN in recent years also have mainly focused on glucose and lipid metabolism disorders and metabolic inflammation, insulin resistance, fibrosis, autophagy and genetics (Thomas et al., 2015; Donate-Correa et al., 2020; Zhongqing et al., 2023), with limited research on endoplasmic reticulum stress and mitochondrial function (Ni and Yuan, 2021). Therefore, it is crucial to clarify the molecular mechanism of ShenQiWan in the treatment of DN for its clinical application. In this study, KKAy mice, a type 2 diabetes model, were fed a high-fat diet to induce severe obesity and diabetes. Our study aims to investigate the specific mechanisms of ShenQiWan in the treatment of DN by observing its effects on renal histopathology in KKAy mice and examining the key factor of endoplasmic reticulum stress, such as Glucose-regulated protein 78 (GRP78), and key factors of mitochondrial fusion, such as Optic atrophy 1 (OPA1), Mitofusin-1 (Mfn1), Mitofusin-2.

## Materials and methods

### Reagents

ShenQiWan (Radix Rehmanniae 24 g, Rhizoma Dioscoreae 12 g, Fructus Corni 12 g, Rhizoma Alismatis 9 g, Poria 9 g, Cortex Moutan 9 g, Ramulus Cinnamomi 3 g, Radix Aconiti Lateralis 3 g), purchased from Beijing Tongrentang; Modified Masson trichrome staining solution (Beijing Suolaibao Technology Co., Ltd., batch number G1345); Glycogen PAS Staining Kit (Beijing Suo Lai Bao Technology Co., Ltd., batch number G1281); Hexaamine silver staining solution (Beijing Regan Biotechnology Co., Ltd., batch number DG0090); mRNA Reverse Transcription Kit (Thermo Fisher Scientific, USA, batch number 32220); MFN1 Polyclonal antibody (Proteintech Corporation, USA, batch number: 13798-1-AP); MFN2 Polyclonal antibody (Proteintech, USA, batch number: 12186-1-AP); anti-OPA1 antibody (Abcam, USA, lot number: ab157457); anti-GRP78 BiP antibody (Abcam, USA, lot number: ab108613).

### Instruments

Blood glucose meter (hanghai Roche Diagnostic Products Co., Ltd.); Automatic biochemical analyzer (Zhongshan Xinrui Medical Equipment Technology Co., Ltd.); Transmission electron microscope (Hitachi, Japan); Synerqy II microplate reader (Bio-Tek, USA); Fluorescence quantitative PCR instrument, vertical electrophoresis system, wet electric transfer printing system (Bole Life Medical Products Co., Ltd.); Gel imager (Protein Simple, USA).

### Animals and treatments

**FIGURE 1:**
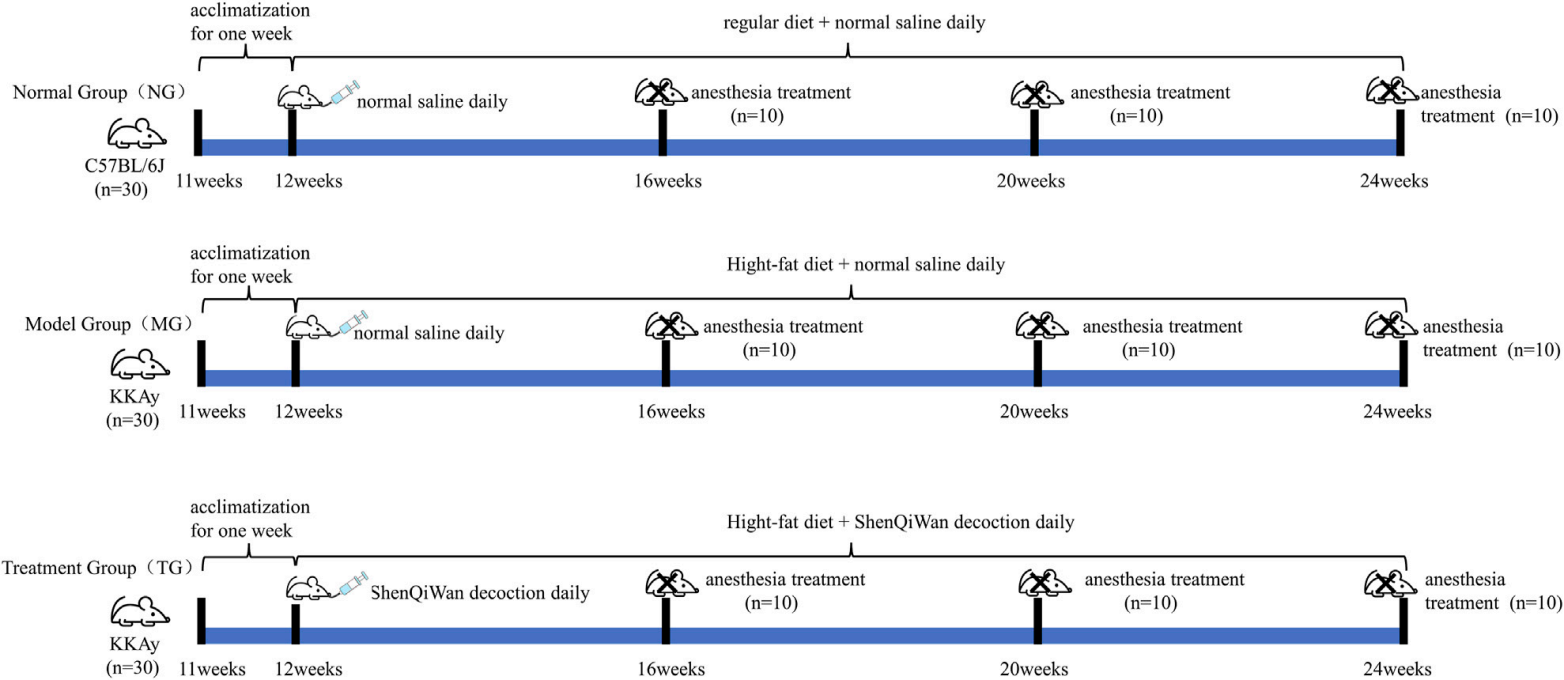
Schematic diagram showing the detal of timeline of drug treatment in KKAy mice.

Sixty 11-week-old male KKAy mice, weighing 30–35 g; thirty 11-week-old male C57BL/6J healthy mice, weighing 25–30 g, were purchased from Beijing Huafukang Biotechnology Co., Ltd. (License No.: SCXK (Beijing) 2014-0004). All experimental mice were fed on the animal experimental platform of the Clinical Research Institute of Beijing China-Japan Friendship Hospital [SYXK (Beijing) 2016-0043], with daily free access to water and food, and the feeding environment was an independent ventilation system within the barrier, room temperature 23°C–25°C, relative humidity 50%–60%. During the whole experiment, KKAy mice were allowed access to a high-fat diet, while C57BL/6J mice were fed a regular diet. All mice were adaptively fed for 1 week, that is, at 12 weeks of age, KKAy mice were screened, grouped, and began to be administered. KKAy Mice with random blood glucose ≥13.9 mmol/L were identified as diabetic mice and randomly divided into the model group (*n* = 30) and the treatment group (*n* = 30), which continued to be fed a high-fat diet. The C57BL/6J mice served as the control group (*n* = 30) and were fed a regular diet. The treatment group received daily oral administration of ShenQiWan decoction (13.5 g/kg), while the model group and the control group received an equal volume of normal saline. Throughout the experiment, all groups of animals had free access to water and food. Ten mice from each group were euthanized at the age of 16 weeks, 20 weeks, and 24 weeks (Figure 1).

### General state of mice and fasting blood glucose testing

The general conditions of the mice were observed daily, including changes in body weight, fur glossiness, changes in diet and water intake, mental status, and status before and after administration. Fasting blood glucose levels were measured every 2 weeks, and mice were fasted for 8 h before blood collection, and blood glucose concentration was measured via tail vein blood sampling method with a blood glucose meter.

### Biochemical analysis

Ten mice were euthanized from each group at 16 weeks, 20 weeks and 24 weeks of age, blood was collected by eyeball blood collection method, and serum and plasma were separated. Total cholesterol (CHOL), triglycerides (TRIG), blood creatinine (CREA), blood urea nitrogen (BUN), and blood uric acid UA in serum were measured by biochemical analyzer, and the level of glycated hemoglobin (HbA1c) in plasma was measured by Elisa kit.

### Urine microalbumin analysis

Urine samples of each group were collected respectively at 16 weeks of age, 20 weeks of age and 24 weeks of age over 24 h, and centrifuged at 3,500 r/min for 10 min and the microalbumin concentrations in urine supernatant were detected by Elisa kit.

### Renal histological analysis

Kidney tissues were fixed with 4% paraformaldehyde solution, and HE staining, PAS staining, PASM staining and Masson staining were performed respectively after wax embedded and sliced. Some kidney tissues were fixed with 2.5% glutaraldehyde solution, and the paraffin sections were observed under a transmission electron microscope (TEM) after processing for dehydration, embedding, sectioning, and staining with uranyl acetate and lead citrate.

### Analysis of GRP78, OPA1, MFN1 and MFN2 mRNA expressions by real-time PCR

Total RNA was extracted from kidney tissues by using Trizol, and the mRNA expression levels of endoplasmic reticulum stress marker GRP78, mitochondrial fusion key factors OPA1, MFN1 and MFN2 were detected strictly according to the operation steps of Real-time PCR kit with β-actin as the internal reference. Primers for PCR (Table 1) were designed and synthesized by Sangon Biotech (Shanghai) Co., Ltd.

**TABLE 1:** Primer sequence for Real-time PCR.

Gene	Forward Primer (5′-3′)	Reverse Primer (5′-3′)
GRP78	GAC​AAC​ACT​GAC​CTG​GAC​ACT​TGG	TCA​GGA​GGA​GAC​ACG​AAG​CAG​AC
OPA1	CTT​ACA​TGC​AGA​ATC​CTA​ACG​C	CCA​AGT​CTG​TAA​CAA​TAC​TGC​G
MFN1	CCA​TCT​TTC​AGG​TCC​CTA​GAT​C	GCT​CCG​TAC​ATA​CTT​AAG​CTG​A
MFN2	TCA​TCA​GGT​TCA​GCG​TCC​TCT​CC	TGA​CCA​CTC​CTC​CGA​CCA​CAA​G
β-actin	GGA​GAT​TAC​TGC​CCT​GGC​TCC​TA	GAC​TCA​TCG​TAC​TCC​TGC​TTG​CTG

### Analysis of GRP78, OPA1, MFN1 and MFN2 protein expressions by Western blot

Total protein was extracted from kidney tissues by ice-cold lysis buffer (RIPA) for 20 min and then centrifuged at 12,000 rpm for 10 min at 4°C. Protein concentrations were collected in a fresh 1.5 mL tube and determined by the bicinchoninic acid assay (BCA) method. SDS-PAGE gels were prepared, and equal amounts of protein samples were loaded for electrophoresis. After that, proteins were transferred to PVDF membranes in an ice-cold buffer via electroblotting for 2 h. The membranes were blocked in 5% skim milk at room temperature for 1 h, and then incubated overnight at 4°C with the primary antibody against GRP78 (dilution 1:5000), OPA1 (dilution 1:5000), MFN1 (dilution 1:5000), MFN2 (dilution 1:5000). After washing with TBST, the membranes were incubated with the secondary antibody (dilution 1:10000)., at room temperature for 1 h. Enhanced chemiluminescence (ECL) was used for imaging and exposure. The images were processed using ImageJ software for grayscale analysis.

### Data and statistical analysis

GraphPad Prism9.0 software was used for data analysis. Numerical data were expressed as mean ± standard deviation (X ± SD). Depending on the normal distribution and homogeneity of variances, either one-way analysis of variance (ANOVA) or non-parametric tests were chosen for comparisons between groups. and *p*-value less than 0.05 (*p* < 0.05) were considered statistically significant.

## Results

### Performance status of mice

The mice in the control group were bright in coat color, more agile in action, and moved freely. However, the mice in the model group exhibited listlessness, reduced activity, slow movement, and a lackluster coat, and significantly increased water intake and urine volume. Compared with the model group, the above conditions of mice in the treatment group were milder, and the body weights were significantly reduced (Table 2).

**TABLE 2:** Fasting blood glucose and weight of each group.

Groups	16 weeks		20 weeks		24 weeks	
	Weight (g)	Fasting blood glucose (mmol.L^-1^)	Weight (g)	Fasting blood glucose (mmol.L^-1^)	Weight (g)	Fasting blood glucose (mmol.L^-1^)
CG	31.44 ± 2.86	5.00 ± 0.46	30.47 ± 2.13	6.39 ± 1.03	31.42 ± 2.48	5.84 ± 0.74
MG	42.49 ± 2.33*	23.06 ± 6.75*	42.95 ± 2.70*	15.29 ± 5.25*	43.27 ± 2.20*	10.70 ± 3.65*
TG	39.50 ± 2.13*^△^	17.13 ± 5.10*^△^	40.48 ± 2.12*^△^	10.61 ± 2.31*^△^	40.94 ± 1.52*^△^	9.89 ± 3.46*

Values are expressed as mean ± SD (*n* = 7). CG, the control group; MG, the model group, and TG, the ShenQiWan group. Compared with CG, *p < 0.05. Compared with MG, ^△^ *p* < 0.05.

### Effects of ShenQiWan on fasting blood glucose and glycated hemoglobin (HbA1c) levels

**FIGURE 2:**
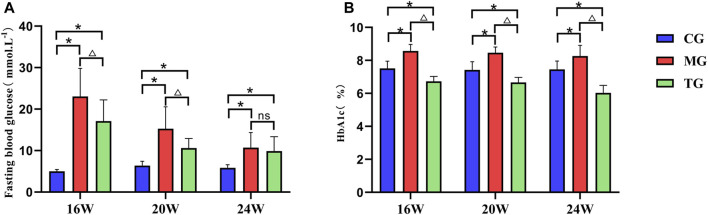
ShenQiWan can reduce blood glucose levels and HbA1c levels of diabetic mice at different weeks of age. **(A)** The change of expression level of fasting blood glucose at different weeks. **(B)** The change of expression level of HbA1c at different weeks. Values are expressed as mean ± SD (*n* = 7). CG, the control group; MG, the model group; and TG, the ShenQiWan group. Compared with CG, *p < 0.05. Compared with MG, ^△^ *p* < 0.05.

The fasting blood glucose levels were significantly higher in the model group compared with those in the control group at all weeks of age, and the differences were statistically significant (*p* < 0.05). The fasting blood glucose levels in the treatment group decreased to varying degrees compared with those in the model group, and the differences from the group at the age of 16 weeks, 20 weeks were statistically significant (*p* < 0.05) (Figure 2A). The results showed that the level of plasma HbA1c in the model group at all weeks of age was significantly higher in comparison with that in the control group, and the level of HbA1c in the treatment group was significantly lower than that in the model group, the differences were statistically significant (*p* < 0.05) (Figure 2B).

### Effects of ShenQiWan on CHOL, TRIG, CREA, BUN and urinary microalbumin levels

**FIGURE 3:**
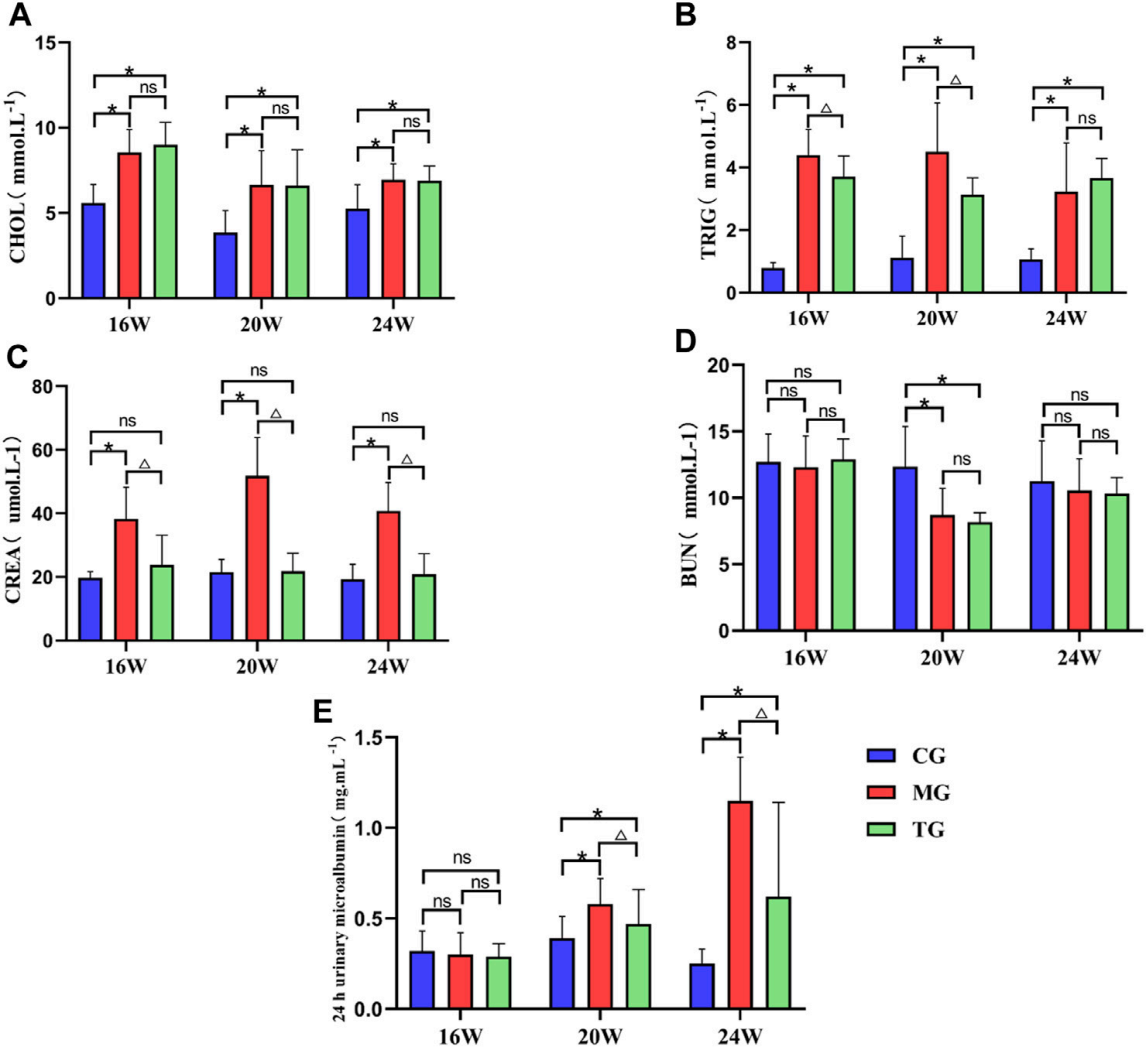
ShenQiWan can reduce blood lipid levels and deterioration of renal function. **(A)** Changes in CHOL levels at different weeks of age. **(B)** Changes in TRIG levels at different weeks of age. **(C)** The change of expression level of CREA at different weeks. **(D)** The change of expression level of BUN at different weeks. **(E)** The change of expression level of urinary microalbumin at different weeks. Values are expressed as mean ± SD (*n* = 7). CG, the control group, MG, the model group; and TG, the ShenQiWan group. Compared with CG, *p < 0.05. Compared with MG, ^△^ *p* < 0.05.

The results showed that the levels of TRIG and CHOL were significantly increased in the model group compared to those in the control group at each week of age (*p* < 0.05) (Figures 3A, B). The level of TRIG was significantly lower in the treated group than that in the control group at the age of 16 weeks and 20 weeks (*p* < 0.05) (Figure 3B). While the level of CHOL in the treatment group was not significantly different from that in the model group at each week of age (*p* > 0.05). (Figure 3A). The level of CREA was significantly increased in the model group compared to that in the control group at each week of age (*p* < 0.05), while it was significantly lower in the treatment group than that in the model group (*p* < 0.05) (Figure 3C). The BUN level in the model group at 20 weeks of age was significantly lower than that in the control group (*p* < 0.05) and had no significant difference compared with that in the treatment group (*p* > 0.05) (Figure 3D). There was no significant difference in urinary microalbumin expression levels of each group at 16 weeks of age (*p* > 0.05). Urinary microalbumin levels were significantly higher in the model groups than those in the control group at 20 weeks of age and 24 weeks of age (*p* < 0.05), while the treatment group showed a significant reduction in urinary microalbumin compared to the model group, with statistically significant differences (*p* < 0.05) (Figure 3E).

### Effects of ShenQiWan on morphological changes in kidney tissues of diabetic mice

**FIGURE 4:**
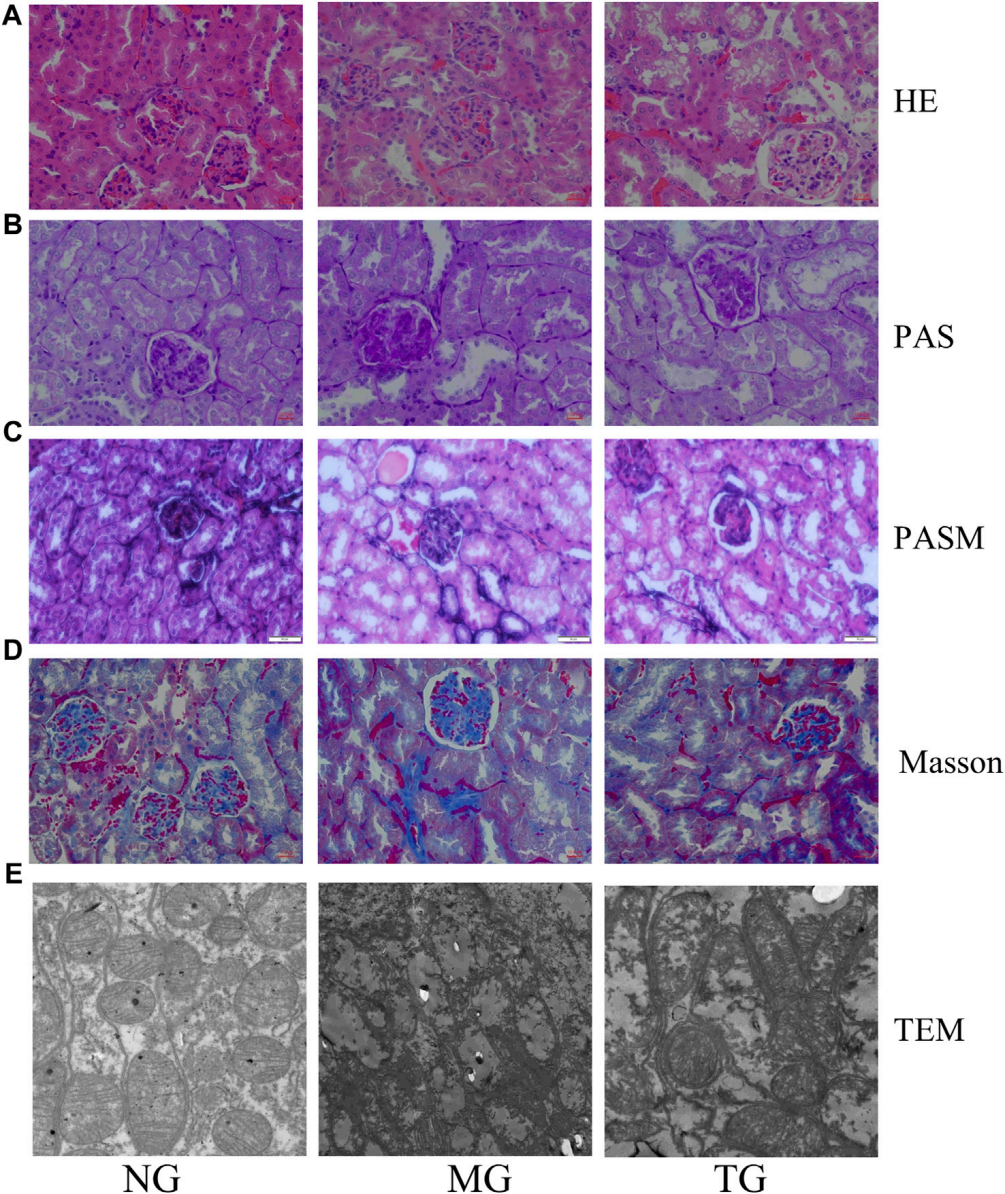
Pathological changes of diabetic mice. HE staining (×40), PAS staining (×40), PASM staining (×40), Masson staining (×40). **(A)** HE staining revealed that ShenQiWan can alleviate changes such as narrowing of renal glomerular tubules, thickening of the renal glomerular basement membrane, swelling of renal tubular epithelial cells, and luminal constriction. **(B)** PAS staining revealed that ShenQiWan can alleviate changes such as collagen fiber proliferation and glycogen deposition in the renal tubular epithelium and stroma. **(C)** PASM staining revealed that ShenQiWan can alleviate changes such as mesangial tissue proliferation and sclerosis, irregular thickening of the capillary wall, partial glomerular sclerosis, and significant tubular atrophy. **(D)** Masson staining revealed that ShenQiWan can also alleviate glomerulosclerosis and interstitial fibrosis. **(E)** TEM observation revealed that ShenQiWan can reverse mitochondrial fragmentation, endoplasmic reticulum swelling and fracture, irregular thickening of the glomerular basement membrane to some degree.

Compared to the control group, the model group showed various morphological changes in the kidney tissues caused by DN: HE staining revealed narrowed glomerular lumens, thickening of the renal glomerular basement membrane, edema and ballooning of renal tubular epithelial cells (Figure 4A). In addition to the above conditions, PAS staining also revealed collagen fiber proliferation and glycogen deposition in the renal tubular epithelium and stroma (Figure 4B). PASM staining revealed mesangial tissue proliferation and sclerosis, irregular thickening of the capillary wall, partial glomerular sclerosis, significant tubular atrophy and uniform red-stained protein in the lumen of the renal tubule (Figure 4C). Masson staining also showed glomerulosclerosis and interstitial fibrosis (Figure 4D) (Supplementary Figure S1). However, treatment with ShenQiWan reversed these changes to some degree (Figures 4A–D). Transmission electron microscopy (TEM) observation revealed that the control group showed the glomerular endoplasmic reticulum, mitochondria and basement membrane with defined structures and normal foot processes. However, the model group showed mitochondrial fragmentation, endoplasmic reticulum swelling and fracture, irregular thickening of the glomerular basement membrane, effacement of foot processes. After treatment, the severity of all of the morphological changes listed above decreased to varying degrees (Figure 4E).

### Effects of ShenQiWan on the mRNA expression levels of GRP78, OPA1, MFN1, MFN2

**FIGURE 5:**
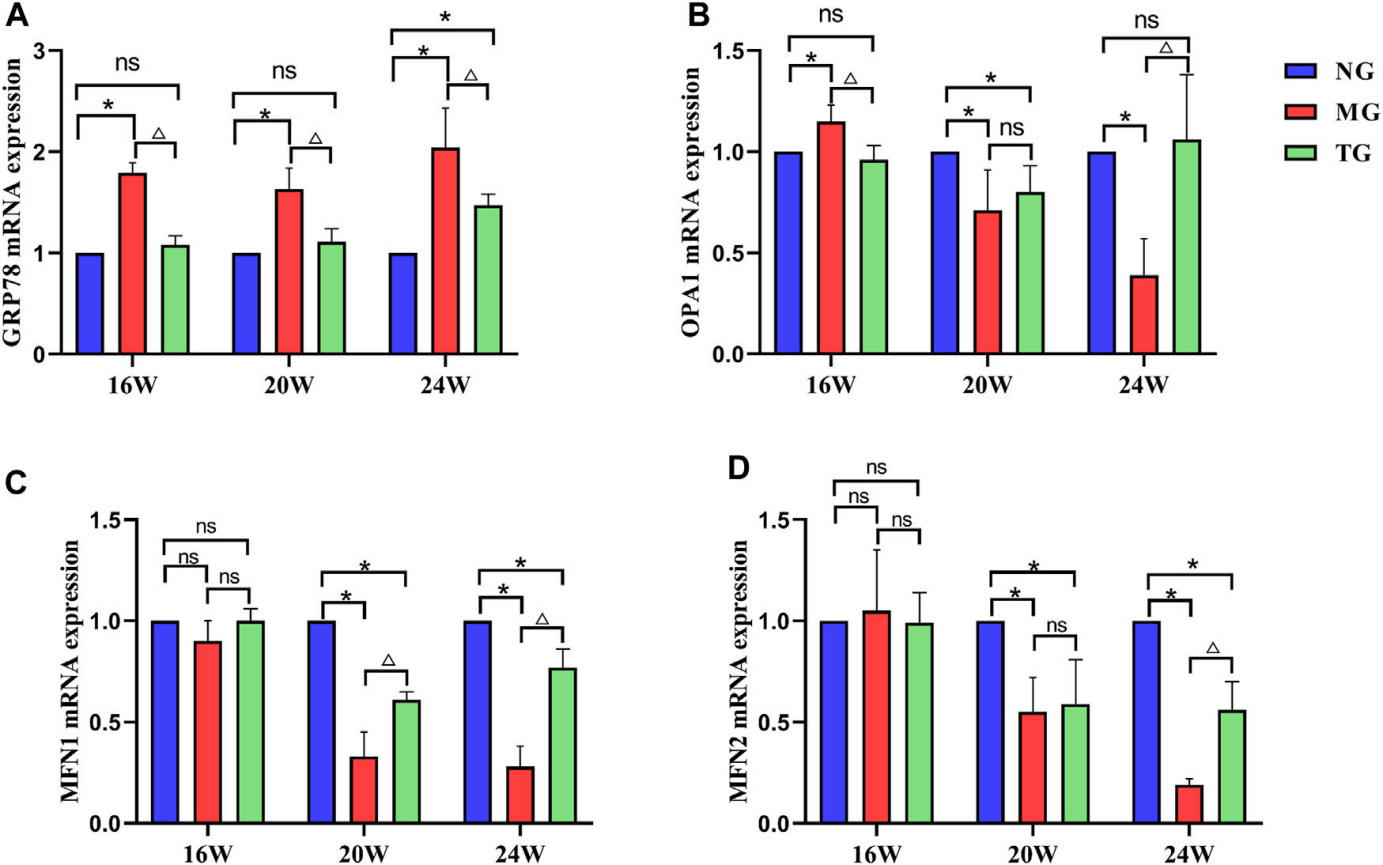
ShenQiWan can influence the expression levels of GRP78, OPA1, MFN1, and MFN2 mRNA. **(A)** ShenQiWan can decrease GRP78 mRNA expression levels. **(B)** ShenQiWan can increase OPA1 mRNA expression levels. **(C)** ShenQiWan can increase MFN1 mRNA expression levels. **(D)** ShenQiWan can increase MFN2 mRNA expression levels. Values are expressed as mean ± SD (*n* = 3). CG, the control group; MG, the model group; and TG, the ShenQiWan group. Compared with CG, *p < 0.05. Compared with MG, ^△^ *p* < 0.05.

Real-time PCR results showed that the relative mRNA expression level of GRP78 in the model group was significantly higher compared with that in the control group at all weeks of age (*p* < 0.05), and the relative mRNA expression level of GRP78 was significantly lower than that in the model group after treatment with ShenQiWan (*p* < 0.05) (Figure 5A). The relative mRNA expression levels of OPA1, MFN1 and MFN2 in the 20-week-old and 24-week-old model groups were significantly lower than those in the control group (*p* < 0.05) (Figures 5B–D), however, the relative mRNA expression levels of OPA1, MFN1 and MFN2 increased to varying degrees after treatment with ShenQiWan, with significant differences observed in the 24-week-old group (*p* < 0.05) (Figures 5B–D).

### Effects of ShenQiWan on the protein expression level of GRP78, OPA1, MFN1, MFN2

**FIGURE 6:**
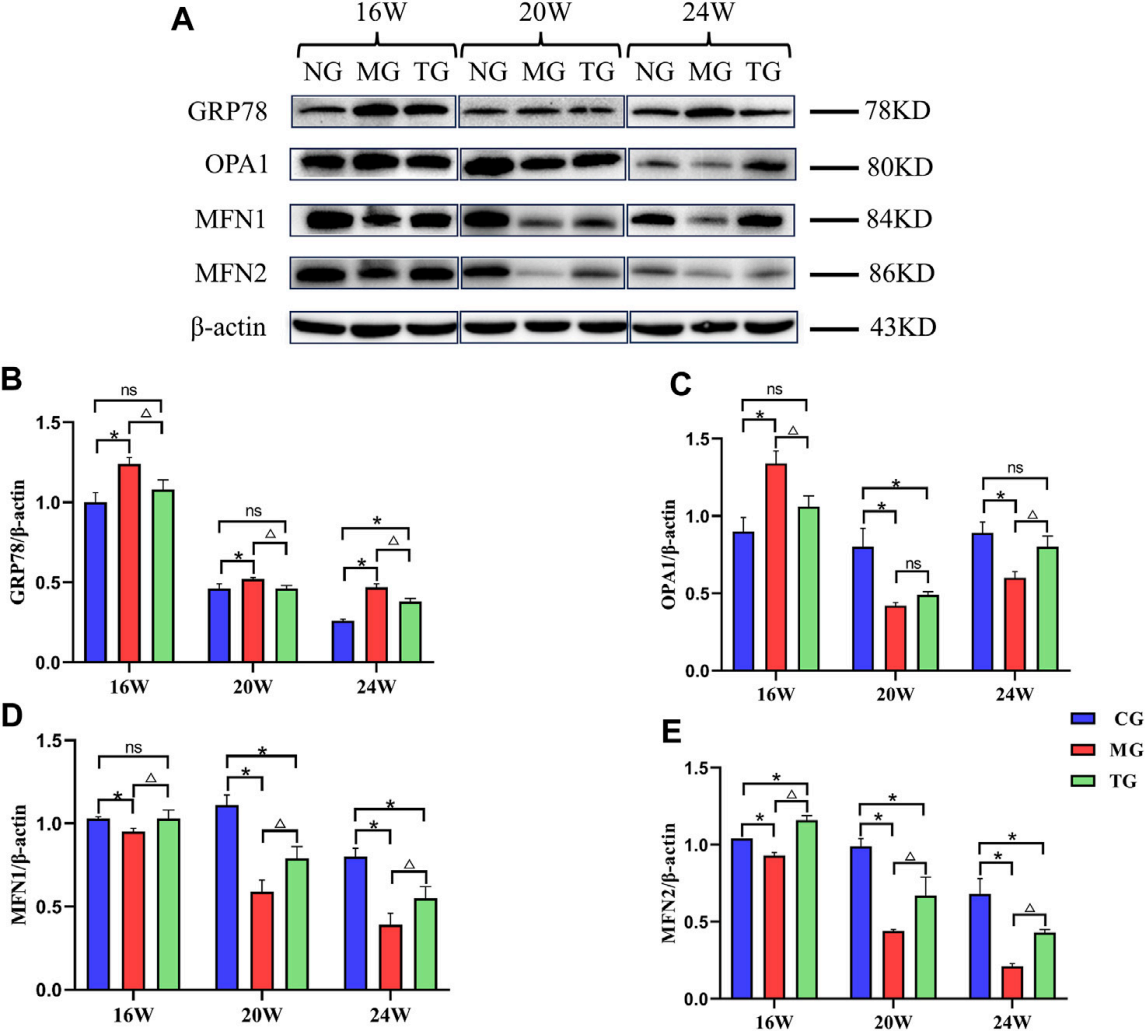
ShenQiWan can influence the protein expression levels of GRP78, OPA1, MFN1 and MFN2. **(A)** Western blot analysis of GRP78, OPA1, MFN1, MFN2. **(B)** ShenQiWan can decrease the protein expression levels of GRP78. **(C)** ShenQiWan can increase the protein expression levels of OPA1. **(D)** ShenQiWan can increase the protein expression levels of MFN1. **(E)** ShenQiWan can increase the protein expression levels of MFN2. Values are expressed as mean ± SD (*n* = 3). CG, the control group; MG, the model group; and TG, the ShenQiWan group. Compared with CG, *p < 0.05. Compared with MG, ^△^ *p* < 0.05.

Protein expressions were measured by Western blotting, using β-actin as a housekeeping gene (Figure 6A). Western blot analysis showed the model group displayed significantly higher levels of GRP78 when compared to levels measured in the control group at all weeks of age, treatment with ShenQiWan significantly inhibited the protein expression of GRP78 (*p* < 0.05) (Figure 6B). The protein expression levels of OPA1 in the 20-week-old and 24-week-old model groups were significantly lower than those in the control group (*p* < 0.05), however, treatment with ShenQiWan reversed decreases in OPA1 protein to varying degrees, and the difference in the 24-week-old group was significant (*p* < 0.05) (Figure 6C). The protein expressions of MFN1 and MFN2 in the model group were significantly reduced compared to those in the control group at all weeks of age (*p* < 0.05), while the treatment group exhibited a significant increase in the protein expression levels of MFN1 and MFN2 compared to those in the model group (*p* < 0.05) (Figures 6D, E).

## Discussion

Diabetic Nephropathy (DN) is one of the most common chronic microvascular complications of diabetes mellitus, which affects approximately 30% of diabetic patients worldwide and may place a heavy economic burden on patients’ families and society in the future (Ruiz-Ortega et al., 2020). Currently, the pathogenesis of DN is not well understood, and there is also a lack of effective therapeutic drugs specifically targeting DN. Therefore, it is particularly important to seek new therapeutic drugs and deeply explore the pathogenesis of DN. In recent years, traditional Chinese medicine has been widely used in the treatment of diabetic nephropathy (Yulong et al., 2021). In this study, ShenQiWan was used to treat KKAy diabetic mice, and the results showed that ShenQiWan could significantly improve kidney damage in KKAy mice, and its mechanism was closely related to mitochondrial-endoplasmic reticulum coupling.

Diabetes falls under the category of “Xiao Ke” syndrome in traditional Chinese medicine. Zhang Zhongjing first used ShenQiWan to treat diabetes in his book “Synopsis of Golden Chamber.” ShenQiWan is composed of Radix Rehmanniae, Chinese Yam, Fructus Corni, Rhizoma Alismatis, Poria cocos, Cortex Moutan, Ramulus Cinnamomi, Paofuzi and has the effect of invigorating the kidney yang. In modern clinical research, ShenQiWan is also often used to treat various diseases caused by liver-kidney yang deficiency (Zhou et al., 2016). After renal damage occurs in diabetic patients, symptoms such as polyuria and proteinuria will occur in the early stage, dominant proteinuria in the late stage, and finally develop into renal failure. The pathological changes in the early stage are mainly glomerular hypertrophy, basement membrane thickening and accumulation of mesangial extracellular matrix. While the progression to the later stage shows fibrosis of the glomerulus fibrosis and tubulointerstitial fibrosis (Chen et al., 2017). KKAy mice, as a spontaneous animal model of type 2 diabetes, are widely used in the study of type 2 diabetes and its complications. In this study, three time points (16 weeks old, 20 weeks old and 24 weeks old) were selected to observe the general state, body weight, fasting blood glucose, relevant biochemical indexes in serum and urine, pathological changes in kindy tissues, as well as the expression of endoplasmic reticulum stress and mitochondrial fusion key proteins in KKAy diabetic model mice. It aims to investigate the protective mechanism of ShenQiWan against kindy injury in diabetic mice.

Our results showed that ShenQiWan significantly improved symptoms such as mental depression, drowsiness, polyphagia and polyuria in diabetic mice. After treatment with ShenQiWan, the body weight and fasting blood glucose of diabetic mice decreased significantly. There was also an improvement in urine microalbumin levels and relevant serum biochemical indicators (serum creatinine, blood urea nitrogen, etc.) to varying degrees. These positive effects were observed at all three time points, indicating that ShenQiWan plays an important role in the entire treatment of diabetic nephropathy. HE, PAS, PASM and Masson staining showed that ShenQiWan could improve the proliferation of glomerular mesangial cells, thickening of basement membrane, glomerulosclerosis, edema of renal tubular epithelial cells, glycogen deposition in renal tissue and interstitial fibrosis in diabetic mice. These findings suggest that ShenQiWan can protect kindy morphology and alleviate kidney damage in KKAy diabetic mice. Transmission electron microscopy observations also showed that the glomerular basement membrane in the model group was thickened and hardened, the podocyte nucleus was swollen, the foot processes were fused and disappeared, the mitochondria were fragmented, the ridges were broken and disappeared, and the endoplasmic reticulum was swollen and broken. However, these pathological changes were alleviated in the treatment group.

The Endoplasmic reticulum (ER) is an important site for protein secretion, synthesis, processing and modification. Stimuli such as high glucose, high lipids, oxidative stress, ischemia, and hypoxia lead to misfolding of proteins in the endoplasmic reticulum, disrupting endoplasmic reticulum homeostasis, and causing endoplasmic reticulum stress (ERS). GRP78 protein is a hallmark factor of ERS, and its upregulation is specific to endoplasmic reticulum stress (Shu et al., 2008). ERS also had been found to play an important role in the development of DN, and can attenuate endoplasmic reticulum stress-induced apoptosis by reducing the expression of GRP78 and PERK (Xiong et al., 2020). Previous results by our group also indicated that type 2 diabetic mice exhibited enhanced ERS and increased expression of related factors such as GRP78, PERK and EIF2α, and traditional Chinese medicine and its main components can alleviate ERS by downregulating the expression of related factors (Zhi-hui et al., 2015; Da et al., 2017; Shujing et al., 2017). Furthermore, our previous research on another diabetic model, the ZDF rat model, also proved that ShenQiWan may alleviate kidney injury in ZDF rats by potentially affecting endoplasmic reticulum stress (Liu et al., 2022). In this study, we further explored the relationship between ShenQiWan and the key factor of endoplasmic reticulum stress, GRP78. Analysis of GRP78 mRNA and protein expression also showed that the expression levels of GRP78 mRNA and protein in the renal tissue of the model group were significantly higher than those in the control group, and the expression levels were also decreased to varying degrees after treatment with ShenQiWan. This study further confirmed the important role of endoplasmic reticulum stress in diabetic nephropathy and also suggested that ShenQiWan can alleviate renal injury in diabetic mice by alleviating endoplasmic reticulum stress. Our study preliminarily elucidates from the perspective of animal experiments that ShenQiWan can reduce kidney injury by affecting cellular endoplasmic reticulum stress. The direct interaction between ShenQiWan and the key endoplasmic reticulum stress factor GRP78, as well as the specific mechanisms of ShenQiWan about endoplasmic reticulum stress, requires further investigation *in vitro* experiments.

Mitochondria are the main sites for intracellular oxidative phosphorylation and synthesis of ATP, providing energy for cellular activities. The states of dynamic fusion and division of mitochondria are prerequisites for their self-repair and function. The function of mitochondria is closely related to their structure, and the intact structure is crucial for mitochondrial activities and energy metabolism. The regulation of mitochondrial structure mainly occurs through fusion and fission processes. Mitochondrial outer membrane fusion is regulated by mitofution1 (MFN1) and mitofution2 (MFN2), and mitochondrial inner membrane fusion is regulated by Opticatrophy1 (OPA1) (Rosselin et al., 2017). OPA1 is a key protein of the mitochondrial inner membrane, which is closely related to the fusion of the mitochondrial inner membrane and maintenance of the mitochondrial ridge structure. In this study, we observed mitochondrial fragmentation and mitochondrial cristae deformation and disappearance in the model group by transmission electron microscopy, and the above phenomena were significantly alleviated after treatment with ShenQiWan. We found that the expressions of OPA1 mRNA and protein in the model group were higher than those in the normal group in the early stage of the disease. However, the expression levels of OPA1 decreased significantly again compared to the control group along with the development of the disease, which increased significantly compared to the model group after treatment with ShenQiWan. We speculate that the initial expression of OPA1 in the early stage may be related to the emergence of stress response in cells. However, in general, the expression of OPA1 mRNA and protein showed a time-dependent downregulation with the development of the disease, which also reflected the important role of OPA1 in the mitochondrial ecological balance.

Mitochondrial outer membrane fusion proteins MFN1 and MFN2 have a high degree of homology, which play a role in promoting mitochondrial fusion at different stages and are essential for maintaining the homeostasis of mitochondrial morphology. MFN1 mainly promotes the combination of mitochondria in the early stage of fusion, while MFN2 mainly plays a role in the late stage of mitochondrial fusion. In this study, analysis of MFN1 and MFN2 mRNA and protein expression showed that, in general, the expression levels of MFN1 and MFN2 in the kidney tissues of the model group were significantly lower than those in the control group, and the expression level was significantly increased after treatment with ShenQiWan. The expression of MFN1 protein in the treatment group at 16 weeks of age was significantly increased compared to that in the model group, with no significant difference compared to the control group. The expression of MFN2 in the treatment group at 16 weeks of age was significantly increased compared with the model group and the control group. While the expression levels of MFN1 and MFN2 protein at 20 and 24 weeks of age were significantly higher than those in the model group, but lower than those in the control group. The above results may reflect that ShenQiWan may be better in the early treatment of renal injury. In summary, OPA1, MFN1, MFN2 mRNA and protein expression analysis shows that mitochondrial fusion dysfunction occurs during the development of diabetes, resulting in abnormal mitochondrial morphology, and ShenQiWan can alleviate the renal injury by stabilizing the mitochondrial fusion process.

There is a close association between mitochondria and the endoplasmic reticulum (ER) in terms of structure and function, with the region of their interaction referred to as mitochondria-associated endoplasmic reticulum membranes (MAMs) (van Vliet and Agostinis, 2018). GRP78 and MFN2 proteins are also located in the structural coupling between mitochondria and endoplasmic reticulum. In particular, MFN2 connects the outer mitochondrial membrane and the ER membrane and accumulates at the sites of structural coupling, together with MFN1 supporting the structure of MAM (de Brito and Scorrano, 2008; Leemand and Koh, 2012). Furthermore, studies have demonstrated that MFN2 and MFN1 were not only involved in the process of mitochondrial fusion but also involved in endoplasmic reticulum stress and the interaction between mitochondria and endoplasmic reticulum. (Filadi et al., 2015; McFie et al., 2016; Wang et al., 2021). Our study confirmed that ShenQiWan could alleviate kindy injury in diabetic mice by regulating the expression of endoplasmic reticulum stress key protein GRP78 and mitochondrial fusion proteins MFN1, MFN2 and OPA1. Whether there is a direct interaction between ShenQiWan and the signaling proteins GRP78, MFN1, MFN2, and OPA1, as well as the specific molecular mechanism of ShenQiWan on endoplasmic reticulum stress and mitochondrial fusion, requires further exploration through *in vitro* cell culture system using silencing or inhibiting specific signaling proteins involved in the cellular damage and mitochondrial function and dynamics. Subsequent studies are necessary to take mitochondria-endoplasmic reticulum structural coupling (MAM) as a point of entry and to further investigate the specific molecular mechanisms of ShenQiWan in ameliorating endoplasmic reticulum stress and mitochondrial dysfunction as well as the key signaling pathways in the process of anti-diabetic kidney injury.

## Conclusion

ShenQiWan can significantly reduce body weight, fasting blood glucose level, urinary microalbumin excretion rate and glycosylated hemoglobin level, improve dyslipidemia and alleviate renal structural damage in diabetic mice at different stages of diabetes nephropathy progression. The underlying mechanism of action may be related to maintaining the stability of mitochondrial-endoplasmic reticulum structure coupling by inhibiting endoplasmic reticulum stress and protecting mitochondrial morphology.

## Data Availability

The raw data supporting the conclusion of this article will be made available by the authors, without undue reservation.
