# Supplementary figures and images for: ShenQiWan ameliorates renal injury in type 2 diabetic mice by modulating mitochondrial fusion and endoplasmic reticulum stress
Source: Front Pharmacol. 2023 Nov 10;14:1265551. doi: 10.3389/fphar.2023.1265551 (PMC10667480; doi:10.3389/fphar.2023.1265551)

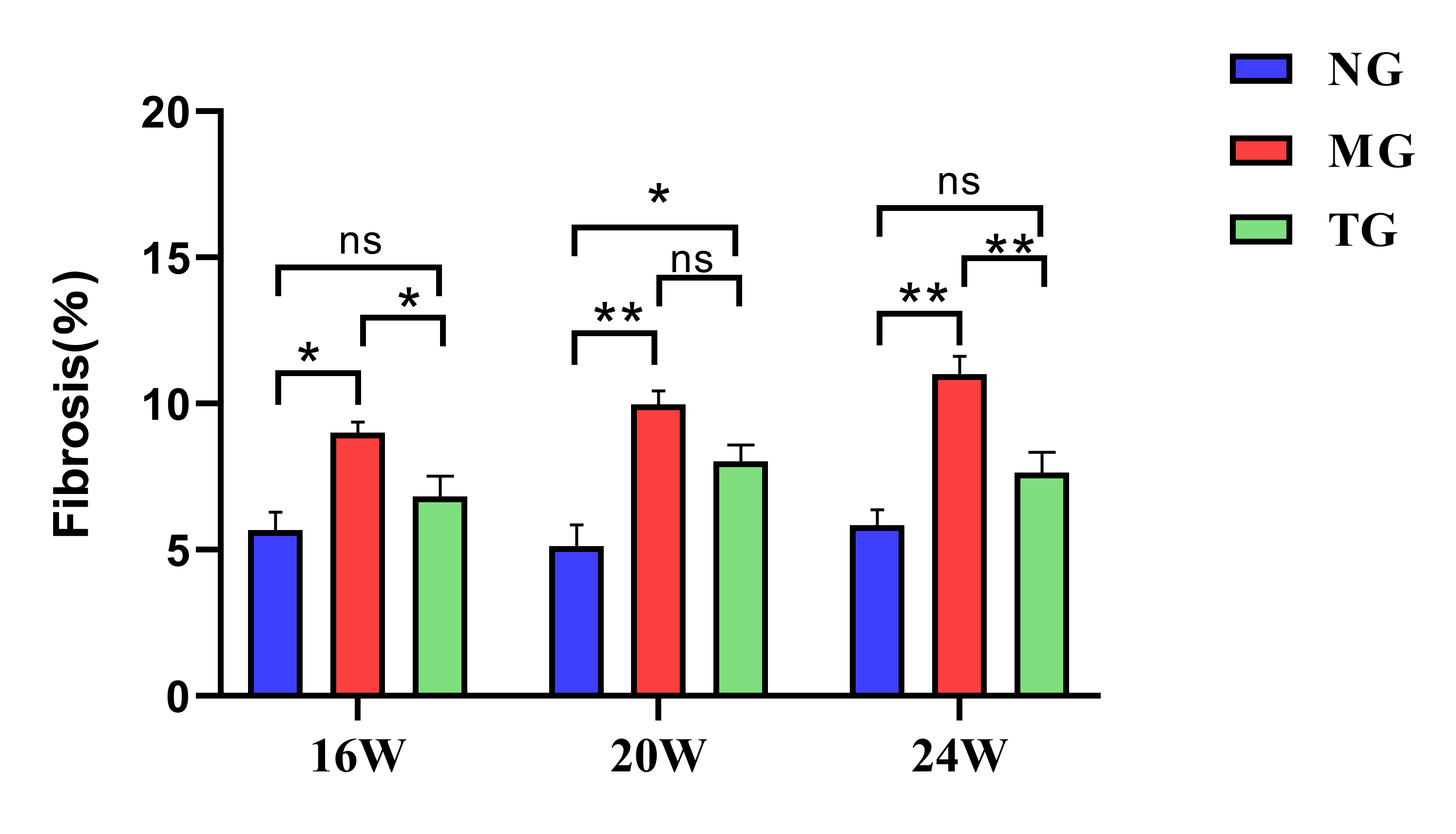

Supplement: Supplementary file 1 [file Image1.TIF]

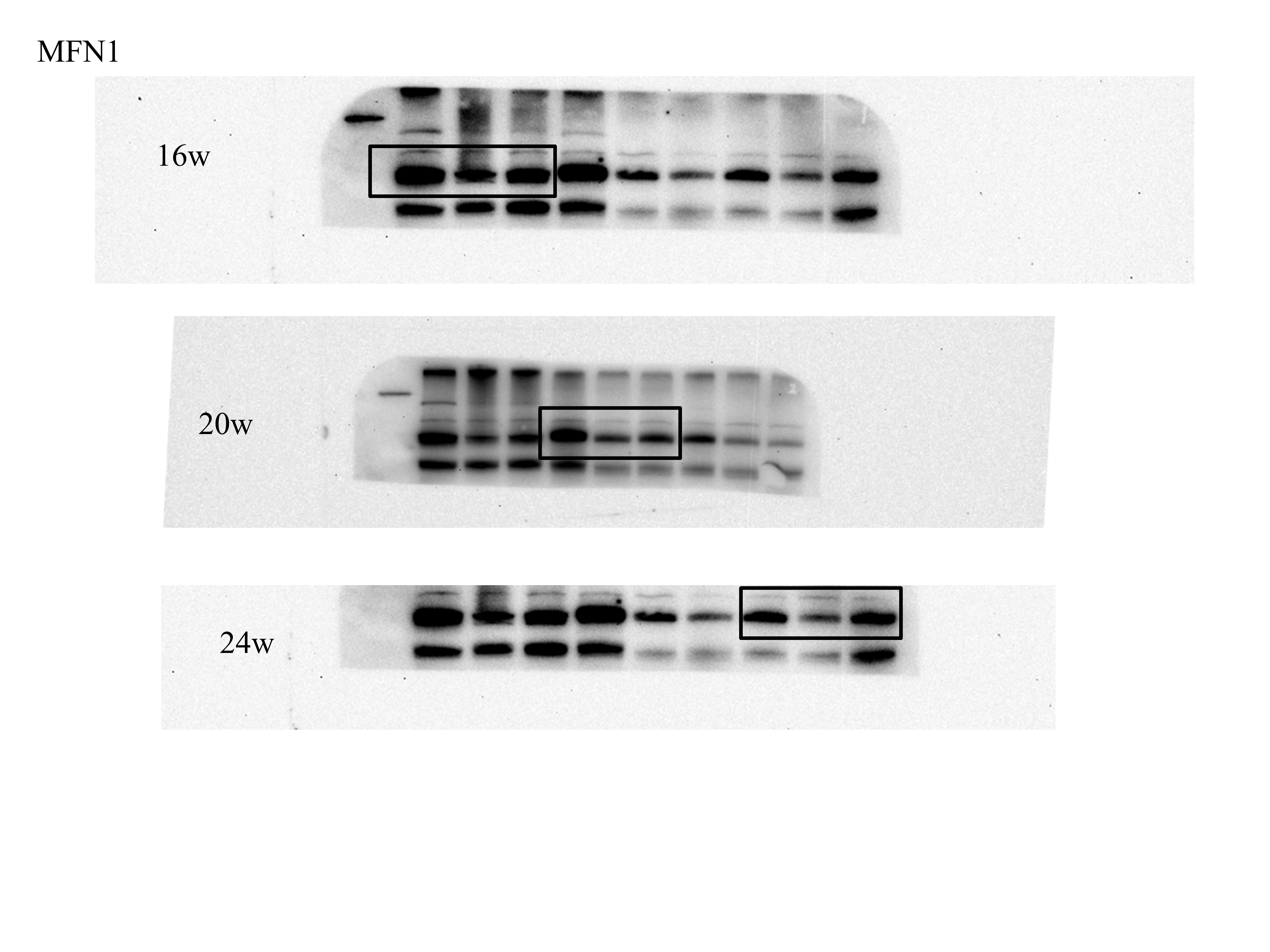

Supplement: Supplementary file 2 [file Image4.PNG]

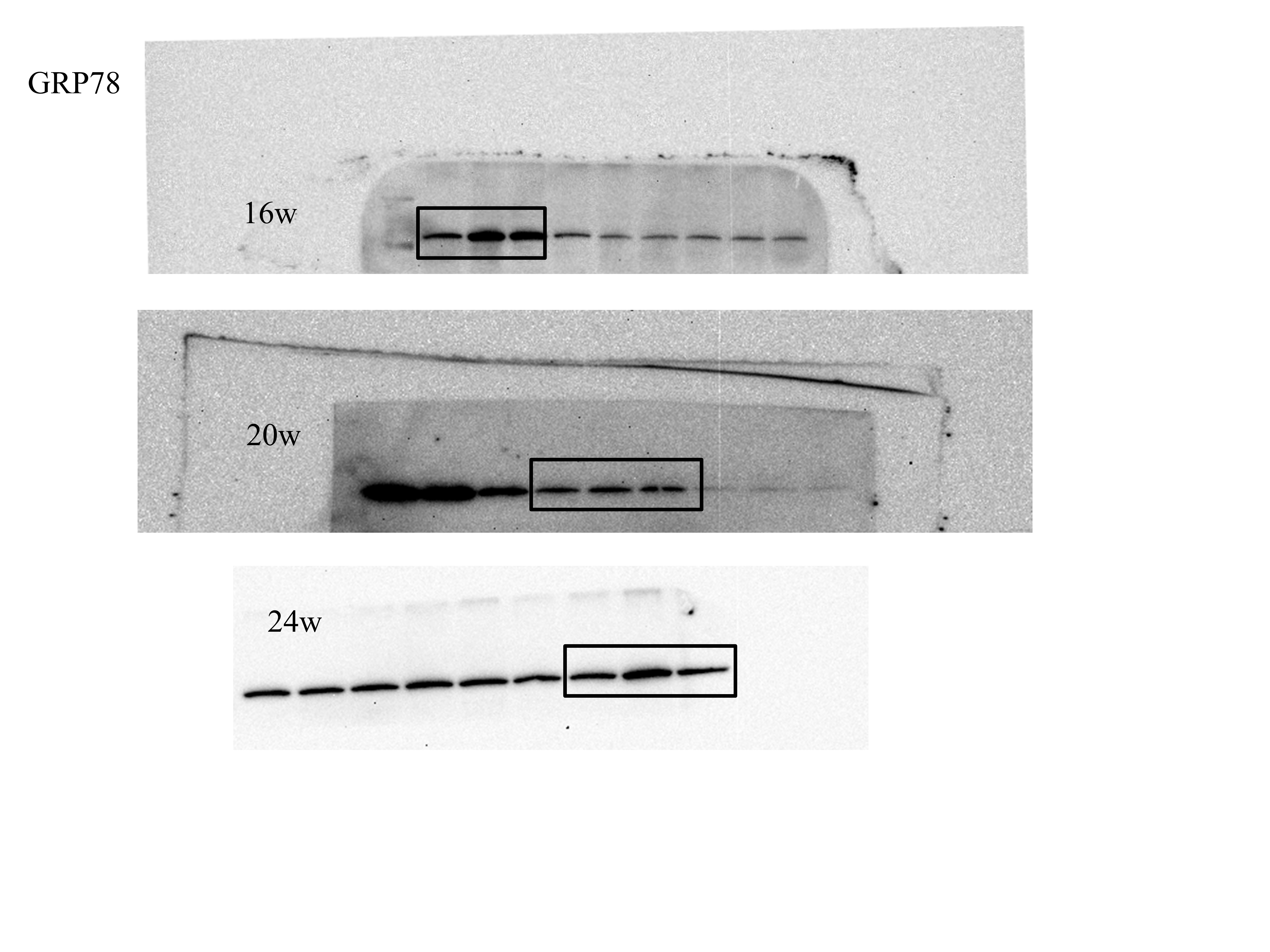

Supplement: Supplementary file 3 [file Image2.PNG]

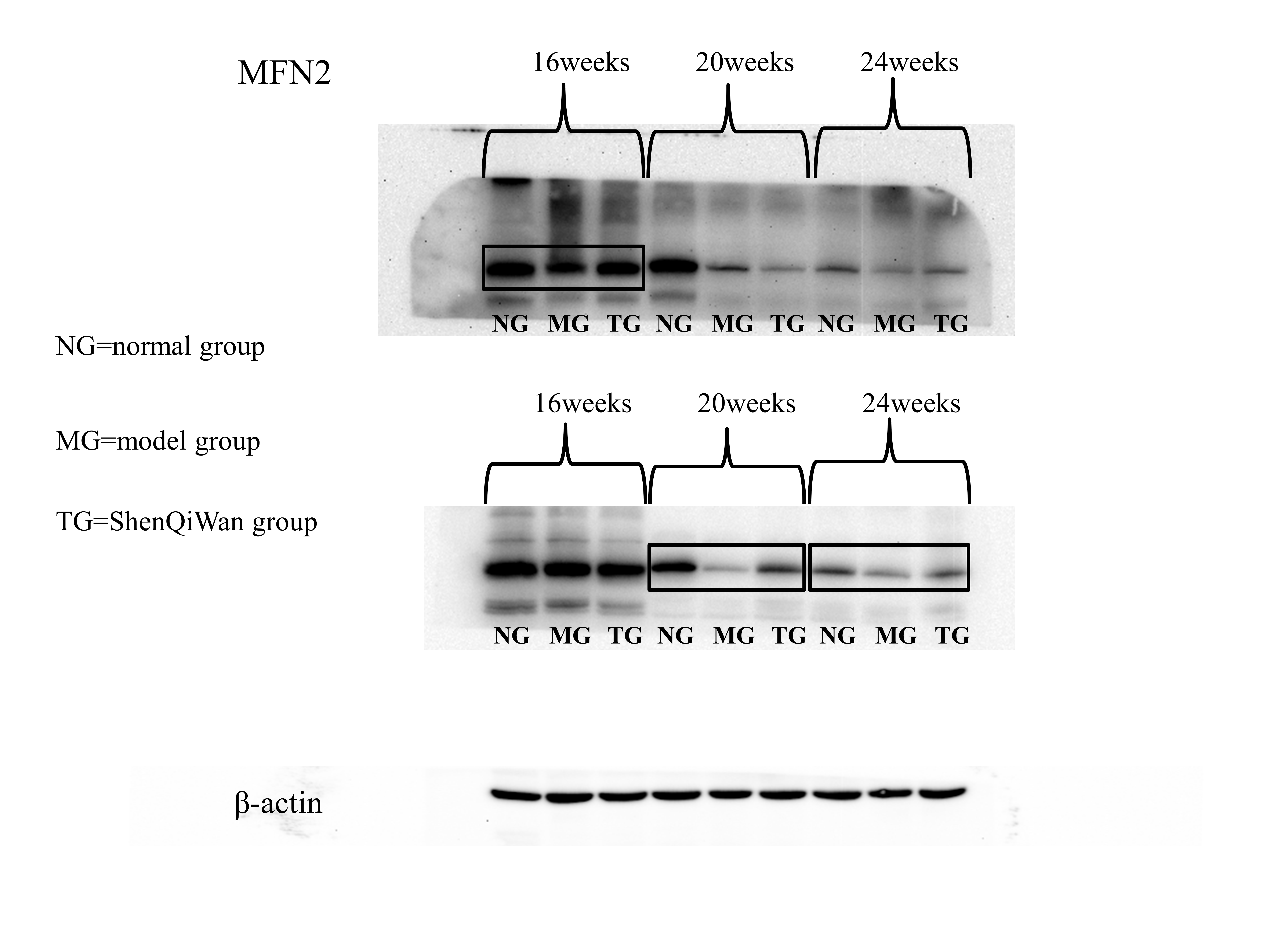

Supplement: Supplementary file 4 [file Image5.TIF]

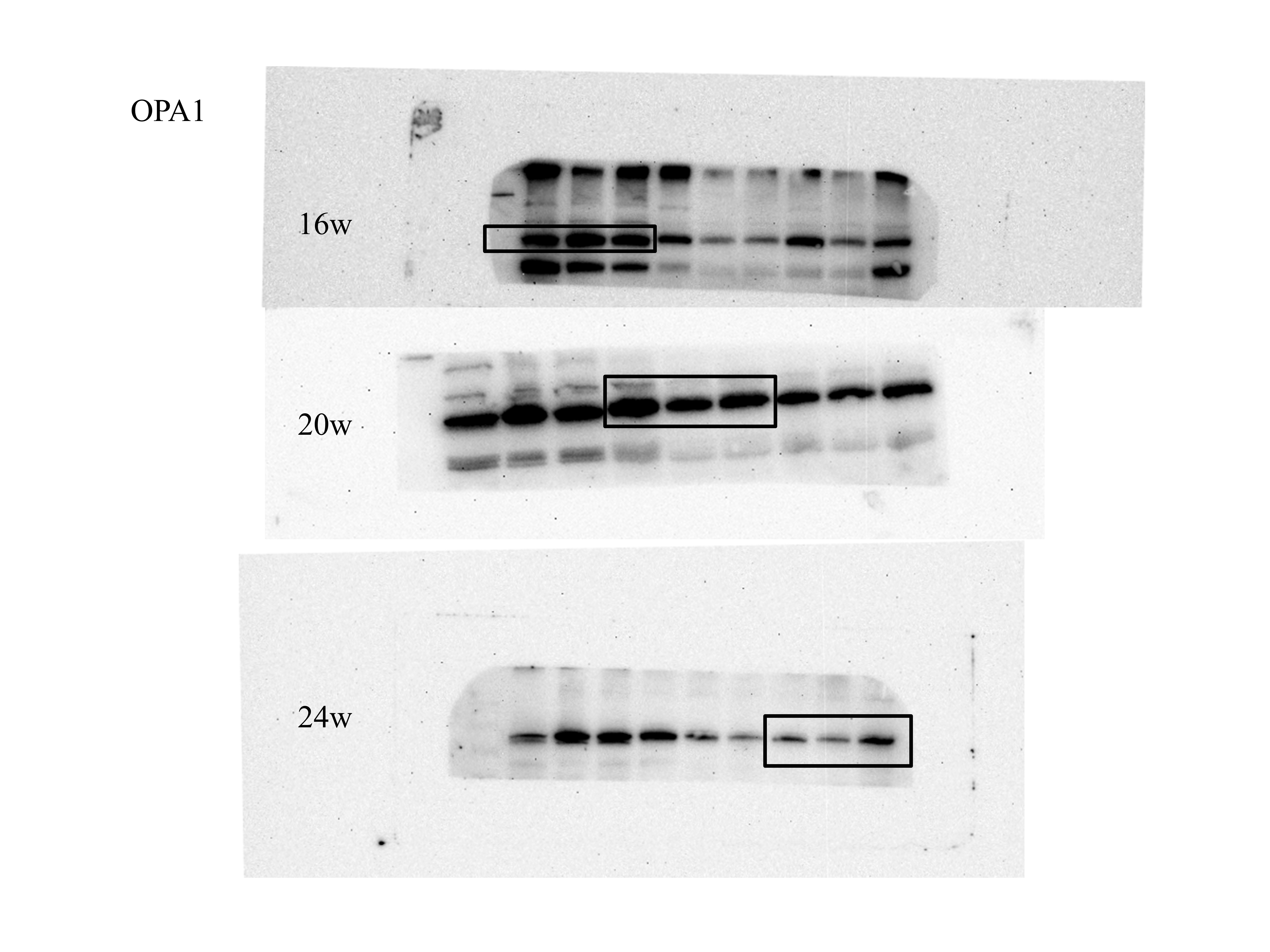

Supplement: Supplementary file 5 [file Image3.PNG]
